# Tobacco smoking in mongolia: findings of a national knowledge, attitudes and practices study

**DOI:** 10.1186/1471-2458-14-213

**Published:** 2014-02-28

**Authors:** Alessandro R Demaio, Jessica Nehme, Dugee Otgontuya, Dan Wolf Meyrowitsch, Palam Enkhtuya

**Affiliations:** 1 Harvard Global Equity Initiative, Harvard Medical School, 651 Huntington Avenue, 02115 Boston, MA, USA; 2 Copenhagen School of Global Health, University of Copenhagen, PO Box 2099, Dk-1014 Copenhagen K, Denmark; 3 National Center for Public Health, Ministry of Health of Mongolia, Ulaanbaatar, Mongolia; 4 Department of Public Health, University of Copenhagen, Øster Farimagsgade 5, P.O. Box 2099, 1014 Copenhagen K, Denmark

**Keywords:** Smoking, Non-communicable diseases, Epidemiology, Mongolia, Asia, KAP, Policy, Health

## Abstract

**Background:**

In 2009, 48% of males aged 15 or over in Mongolia consumed tobacco, placing Mongolia among the countries with the highest prevalence of male smokers in the world. Importantly, tobacco use is one of the four major risk factors contributing to the global burden of non-communicable diseases (NCDs) – the leading cause of mortality in Mongolia. However, the knowledge, attitudes and practices of the Mongolian population with regards to smoking are largely unmeasured. In this context, a national NCDs knowledge, attitudes and practices survey focusing, among other things, on NCD risk factors was implemented in Mongolia in late 2010 to complement the previous WHO STEPwise approach to Surveillance Survey (STEPS) findings from 2009. This publication explores the smoking-related findings of the Knowledge, Attitudes and Practices Survey (KAPS).

**Methods:**

A nationally representative sample size was calculated using methodologies aligned with the WHO STEPS surveys. As a result, 3450 people from across Mongolia were selected using a multi-stage, random cluster sampling method from permanent residents aged between 15 and 64 years. The KAP survey questionnaire was interviewer-administered on a door-to-door basis.

**Results:**

In Mongolia at 2010, 46.3% of males and 6.8% of females were smokers. This practice was especially dominant among males and urban dwellers (MOR 2.2), and more so among the middle-aged (45–54) (MOR 2.1) while still displaying a high prevalence among Mongolian youth (15.5%). The probability of smoking was independent of the level of education. Although the level of awareness of the health hazards related to tobacco smoking was generally very high in the population, this was influenced by the level of education as more people with a primary and secondary level of education believed that smoking at least one pack of cigarette per day was required to harm one’s health (MOR 5.8 for primary education and 2.5 for secondary). Finally, this knowledge did not necessarily translate into a behavioural outcome as 15.5% of the population did not object to people smoking in their house, and especially so among males (MOR 4.1).

**Conclusion:**

The findings of this KAP survey corroborate the 2009 WHO STEPS Survey findings with regards to the prevalence of tobacco smoking in Mongolia. It identifies males, urban dwellers and Mongolian youth as groups that should be targeted by public health measures on tobacco consumption, while keeping in mind that higher levels of awareness of the harms caused by tobacco smoking do not necessarily translate into behavioural changes.

## Background

Tobacco consumption kills almost 6 million people each year, and is expected to kill 8 million per year by 2030 if the current trend of increasing global tobacco consumption remains unaddressed [1]. Of these deaths, 600 000 are attributable to second-hand smoke only [1]. Moreover, most of these deaths occur in low and middle-income countries (LMICs) where over 80% of the world’s one billion smokers resides [1]. One of these middle-income countries, Mongolia, is particularly burdened by a high prevalence of tobacco consumption that is contributing to the increase of non-communicable diseases, the leading cause of death in the country.

Indeed, in 2009, 48% of males aged 15 or over in Mongolia used tobacco, placing Mongolia among the countries with the highest prevalence of male smokers in the world [2]. This contrasts with only 6.9% of Mongolian women of corresponding ages using tobacco in the same year [2]. It is important to look at the Mongolian situation with regards to tobacco control policies and regulations in the years preceding this KAP study. First, as a step in identifying gaps that might contribute to the high prevalence of tobacco consumption in Mongolia and second, as a reference to contextualise our research results and suggest other or new public health measures.

The first policy document relating to tobacco control in Mongolia, the Law of Mongolia to Combat Tobacco Hazards, appeared in 1993 under the leadership of the Minister of Health and with the support of the World Health Organisation (WHO) [3]. The WHO Framework Convention on Tobacco Control (WHO FCTC) was ratified in January 2004 [4]. Furthermore, the Law of Mongolia on Tobacco Control and the National Programme on Non-Communicable Disease Control was adopted in 2005 [3]. This law contains a number of broad statements concerning the promotion of research and the exchange of information on tobacco control and includes a provision on the establishment of an integrated system of tobacco surveillance [3].

More concretely, these policies and regulations signify a number of things. As of December 2010, public transport was mandated to be smoke-free, but it was legal to smoke in other public spaces. Furthermore, sub national powers (21 *aimags* or provinces) do not have the right to implement smoke-free laws [4].

Picture health warnings on tobacco packages, which have been shown to be effective in reducing tobacco consumption, are mostly compliant with the FCTC recommendations as they take up to 33% of the bottom-front and bottom-back of packages [1,4,5].

Bans on tobacco advertisement, promotion and sponsorship in Mongolia are detailed in three laws: the Law on Tobacco Control, the Law on Advertisement, and the Law on Public Radio and Television [3]. Direct bans are widespread, but the country shows a medium compliance with such bans as per a WHO compliance score. Indirect bans are similarly widespread, except for using brand names of tobacco products on non-tobacco products, but the country similarly shows a relatively low compliance score with such bans [4]. This low compliance is reflected in the fact that the tobacco industry has been engaging in advertisement, promotion and sponsorship activities through all communication channels in Mongolia [3].

Although the sale of tobacco is banned for Mongolians under the age of 16 years, it appears that the clear indication of this law at point of sales is not sufficiently guided by national policies. National policies are moreover failing to clearly prohibit the direct availability of such tobacco products [3].

In terms of tobacco taxation, the excise tax on tobacco has been increasing since 1995, and 2% of it is legally required to be directed towards funding tobacco control measures [3]. However, there was no increase in taxation of the lowest cost brand between 2008 and 2010 at least, while there was a 7% increase in taxation of the most popular brand [4].

Finally, good support and treatment options are unavailable for patients addicted to smoking. Indeed, there is no toll-free phone service for smokers who are considering quitting, and few places offer support for smoking cessation counselling [4]. Costs related to such support are moreover not covered by insurance, except partially in hospitals [4]. Nicotine replacement therapies are available without prescription, however their cost is not covered, and no pharmacological alternative with proven effectiveness are sold in the country [4].

In summary, national policies for tobacco control in Mongolia appear to be compliant with the WHO Framework Convention on Tobacco Control. However, no quantitative evidence of their impact is available. In the context of an epidemic of non-communicable diseases, it is relevant to address the knowledge, attitudes and practices of Mongolian people with regards to smoking, and thus better inform national and regional public health policy actions. This KAP survey aimed to explore four domains:

1. The prevalence of current smokers of tobacco and practices surrounding tobacco smoking

2. Knowledge and attitudes regarding the risks of tobacco smoking

3. Knowledge and attitude regarding the risks of second-hand smoking

4. Healthcare counselling on tobacco smoking.

## Methods

### Setting and population

A door-to-door, household-based questionnaire was conducted on a nationally-representative sample. The full study protocol has been published [6].

### Sample size

A nationally-representative sample was estimated to allow disaggregated data analysis according to gender, urbanicity, level of education and other key demographics. It was calculated considering a 95% confidence interval. A design effect was adopted, based on methodologies used for the WHO STEPS. Assuming a non-response rate of 10%, final sample size was estimated at 3,854.

Of note, the WHO STEPwise approach to Surveillance (STEPS) of risk factors is a standardized method for collecting, analyzing and disseminating data for chronic disease risk factors in WHO member countries, whereby small amounts of useful information are collected on a regular and continuing basis to detect trends over time by age and sex [7].

### Sampling methods

Participants were recruited from households across 42 locations in Mongolia (21 urban and 21 rural sites) using a multi-stage, random cluster sampling method. Proportional population to size methods were used for primary and secondary sampling units as well as for simple random sampling from household lists for tertiary sampling units. Finally, a Kish method was used for participant selection within households.

Only permanent residents of the households sampled were eligible for recruitment and had to be aged between 15 and 64 years. In total, 3540 participants completed the survey.

While this survey does not sample the same participants as the 2009 WHO STEPS survey, both methodologies are aligned, and both nationally-representative samples are from the same broader population.

### Questionnaire

Trained, Mongolian field workers from the National Institute for Public Health administered this questionnaire, which explored a range of NCD and risk factor domains, including tobacco smoking.

Of the 106 questions, 13 explored tobacco smoking. All questions regarding tobacco smoking were close-ended.

### Definitions of the KAP survey domains

#### Prevalence of current smokers of tobacco and practices surrounding tobacco smoking

Smoking status was self-reported. Current smokers of tobacco were reported as those having answered positively to the following statements: “I am currently a daily smoker” and “I am a current smoker, but not every day”. This case definition of a current smoker was based on the criteria of the STEPS survey as to align with and triangulate previous national epidemiological findings.

#### Knowledge and attitudes regarding the risks for self of tobacco smoking

This is the largest KAP domain for this topic as 8 of 13 questions addressed risk perception of tobacco smoking. Participants were asked whether they thought that tobacco smoking affects their health as well as how harmful this effect is as indicated on a Likert scale (0/not harmful, 1/moderately harmful, 2/harmful, 3/very harmful). Then, participants were probed as to whether there is a correlation between the amount of cigarettes smoked and the effect on health (any amount of smoking /smoking at least once per week/only daily smoking/smoking more than one pack per day). Participants were specifically questioned about the health effects of tobacco smoking on the lungs and on the heart.

#### Knowledge and attitudes regarding the risks of second-hand smoking

Three questions probed the participants’ risk perception of second-hand smoking. The first assessed basic knowledge about whether or not second-hand smoke can affect the health of others. The second addressed the importance of a smoke-free workplace as indicated on a Likert scale (1/Not at all, 2/Moderately, 3/Important, 4/Very important). One question probed the participants’ attitudes towards second-hand smoke in their homes.

#### Healthcare counselling on tobacco smoking

Participants were asked if a healthcare worker had ever talked to them about the harms of smoking.

### Quality control

In order to maximize scientific rigour in this research, a number of processes were adopted in the questionnaire methodologies. These are outlined in detail in the previously published protocol [8], and include:

1. Translation and back translation

2. Peer and expert review

3. Pretesting using Cognitive Interviewing

4. Piloting

### Analysis

Statistical Package for the Social Sciences (IBM SPSS 20.0.0 Statistics) was used for data entry and analysis. Univariate and multivariate analysis were applied with outcomes for prevalence, confidence intervals, multivariate odds ratios, and p-values. Multivariate Odds Ratios (MOR) were adjusted for gender, urbanicity, age, educational background and employment status.

### Limitations of methods

It should be recognized that the findings of this KAP survey might underestimate the true prevalence of tobacco smoking in Mongolia, first through report (self-report) bias, but also through courtesy bias. Indeed, although interviewers were local and Mongolian-speaking, they were for the most part doctors or government workers, and as such participants might have felt inclined to conceal or minimise unhealthy behaviours. This effect was minimised through the use of a script, in which concepts were explained and questions posed in an identical fashion for each participant.

Of note, non-responders formed around 10% of the sample. The characteristics of the non-responders were not collected, but they are unlikely to affect the outcome given the large sample size and the small percentage of non-responders in question.

Another way that this survey might underestimate the real burden of tobacco consumption in the Mongolian population is its limitation to tobacco smoking as opposed to encompassing all forms of tobacco consumption.

Further, it must be emphasised that this survey was not designed as a stand-alone epidemiological tool. It was instead designed as a KAP tool to align with, and triangulate the findings of the WHO STEPS surveys, for more accurate interpretation and policy development. In this light, it must also be specified that this study was not designed to directly address current policies and regulations regarding tobacco smoking in Mongolia. Rather, this data is to be used as an indirect measure of their impact on the Mongolian population’s knowledge around the health impacts of tobacco smoking and on some aspects of their attitudes and practices towards tobacco smoking.

Finally, this research did not explore knowledge or attitudes around issues such as pro and anti-tobacco messaging, tobacco product labelling and packaging, or tobacco pricing. This was in part due to the need for brevity and reducing the risks of respondent fatigue, but will be the focus of future research.

### Ethics approval

This study was conducted according to the principles of the Helsinki declaration. The Mongolian National Ministry of Health’s Medical Ethical Committee approved the study on the 06 October 2010.

Consent was inferred if participants agreed to be interviewed. Participants were informed of the methods and use of data as well as its anonymous and confidential nature. Participation in the study was voluntary and no financial remuneration was provided.

## Results

Of the 3,854 participants sampled, 3,450 agreed to take part in the study and were included in the analyses (89.5%). Approximately 60% of participants were women, while 50% were from urban areas Table 1.

**Table 1:** Descriptive information on sample population, disaggregated by age, sex, urbanicity, educational level and employment status; Mongolia, 2010

		**Male**	**Female**
	**n (%)**	**n (% )**	**n (%)**
**Total**	3450 (100.0)	1413 (42.0)	2037 (58.0)
**Age (n = 3450)**			
15-24	1100 (28.0)	506 (27.9)	594 (28.0)
25-34	721 (24.3)	280 (25.8)	441 (23.1)
35-44	630 (23.0)	234 (22.2)	396 (23.6)
45-54	507 (19.2)	196 (18.7)	311 (19.7)
55-64	492 (5.5)	197 (5.4)	295 (5.6)
**Location (n = 3450)**			
Urban	1737 (50.3)	702 (49.6)	1035 (50.2)
Rural	1713 (49.7)	711 (50.4)	1002 (49.8)
**Education (n = 3450)**			
Primary or less	219 (6.4)	107 (7.5)	112 (5.4)
Secondary School	2088 (60.5)	919 (65.0)	1169 (57.5)
Tertiary Schooling	1143 (33.1)	387 (27.4)	756 (37.1))
**Employment (n = 3425)**			
Student	717 (20.8)	330 (23.7)	387 (18.8)
Employed	1503 (43.6)	696 (49.9)	807 (39.5)
Unemployed	508 (14.7)	204 (14.6)	304 (14.8)
Retired/Home	697 (20.2)	167 (11.8)	530 (25.9)

All percentages are percentages of the total study population (n = 3450).

The median age of participants was 33 years. No significant difference was found between the median age of rural and urban participants (32 and 34 years respectively, p = 0.08), while female median age was 3 years higher than male median age (p < 0.05).

Regarding education, 6% of participants received a primary school education only, while approximately two-thirds were educated to a secondary school level and 30% had been to university. More urban participants had received secondary or tertiary education, with 2% having received primary education in urban areas as opposed to 11% in rural ones. Moreover, about a fifth of participants were students, half of which were women.

Approximately 40% of participants reported being employed, while 15% were currently unemployed. One-fifth of participants were retired home-makers, of which 75% were women. Finally, there were no significant differences in relation to employment status between rural and urban populations.

### Domain 1: prevalence of current smokers of tobacco

This survey revealed that 46.3% of males and 6.8% of females were current smokers of tobacco (MOR 14.8, p < 0.01), confirming the findings of the 2009 STEPS survey (Table 2) [2].

**Table 2:** Prevalence of current smokers of tobacco within the Mongolian population; 2010

	**Prevalence**	**CI (95%)**	MOR**	**p-value**
**Gender**				
Female	6.8%	(5.7-7.9)	1.0	-
Male	46.3%	(43.7-48.9)	14.8 (11.9-18.4)	<0.01
**Urbanicity**				
Rural	18.4%	(16.6-20.2)	1.0	-
Urban	27.5%	(25.4-29.6)	2.2 (1.8-2.7)	<0.01
**Age**				
15-24	15.2%	(13.1-17.3)	1.0	-
25-34	26.8%	(23.6-30.0)	1.9 (1.3-2.6)	<0.01
35-44	27.1%	(23.6-30.6)	2.0 (1.4-2.9)	<0.01
45-54	28.5%	(24.6-32.4)	2.1 (1.5-3.1)	<0.01
55-64	23.3%	(19.6-27.0)	1.6 (1.1-2.4)	<0.01
**Education**				
Primary or less	25.1%	(19.4-30.8)	1.0	-
Secondary School	22.9%	(21.1-24.7)	0.9 (0.6-1.4)	0.93
Tertiary Schooling	22.6%	(20.18-25.02)	0.8 (0.5-1.2)	0.79
**Employment**				
Student	12.1%	(9.7-14.5)	1.0	-
Retired/Home	17.4%	(15.5-19.3)	2.1 (1.4-3.3)	<0.01
Unemployed	29.3%	(20.6-28.0)	3.2 (2.1-4.8)	<0.01
Employed	28.3%	(25.0-31.6)	2.2 (1.5-3.2)	<0.01

**Multivariate Odds Ratio (MOR) adjusted for gender, urbanicity, age, educational background and employment status.

The prevalence of smoking was lowest in the youngest age group 15–24 years, but increased with age, peaking for participants aged 45–54 years (MOR 2.1) and declining again among the eldest (55–64 years) (Table 2).

Tobacco smoking was furthermore associated with urban dwelling (MOR 2.2, p < 0.01). Although results of the univariate analysis revealed a statistically significant higher prevalence of smoking among the least educated, this difference did not remain significant after adjusting for confounding in the multivariate regression analysis.

Finally, participants were more likely to smoke if they were employed (MOR 2.2, p < 0.01) or unemployed (MOR 3.2, p < 0.01) as opposed to retired or studying.

### Domain 2: knowledge and attitudes regarding the risk for self of smoking tobacco

Approaching 100% of participants acknowledged that smoking affects their health (data not shown).

In multivariate analysis, thinking that at least 1pack/day of cigarette smoking is required to harm health was significant for participants with a primary or secondary level of education (Primary Education MOR 5.8, p < 0.01 Secondary education MOR 2.5, p < 0.01) (data not shown).

In multivariate analysis, thinking that any amount of smoking could constitute harm to health was significant for the participants with a tertiary education (MOR 2.5, p < 0.01) and for the 15–34 year-old (15–24 age group: MOR 0.6, p = 0.01; 25–34 age group: MOR 0.6, p < 0.01). Findings were borderline significant for the employed (MOR 1.4; CI: 1.0-2.0, p = 0.05) Table 3.

**Table 3:** Awareness that any amount of smoking is a harm to health, among Mongolians; 2010

	**Prevalence**	**CI (95%)**	MOR**	**p-value**
**Gender**				
Female	84.1	82.5-85.7	1.0	-
Male	81.3	79.3-83.3	1.2 (1.0-1.4)	0.09
**Urbanicity**				
Rural	82.4	80.6-84.2	1.0	-
Urban	83.5	81.8-85.3	1.0 (0.8-1.2)	0.9
**Age**				
15-24	78.4	76.0-80.8	0.6 (0.4-0.9)	0.01
25-34	82.6	79.8-85.4	0.6 (0.4-0.8)	<0.01
35-44	85.8	83.1-88.5	0.8 (0.5-1.1)	0.2
45-54	85.3	82.2-88.4	0.8 (0.5-1.2)	0.2
55-64	86.9	83.9-90.0	1.0	-
**Education**				
Primary or less	77.7	72.2-83.2	1.0	-
Secondary School	81.1	79.4-82.8	1.3 (0.9-1.0)	0.1
Tertiary Schooling	89.8	88.2-91.7	2.5 (1.6-3.8)	<0.01
**Employment**				
Student	77.0	73.9-80.1	1.0	-
Retired/Home	84.3	81.6-87.0	1.1 (0.8-1.6)	0.6
Unemployed	80.3	76.8-83.8	1.1 (0.8-1.5)	0.7
Employed	86.2	84.5-87.9	1.4 (1.0-2.0)	0.05

**Multivariate Odds Ratio (MOR) adjusted for gender, urbanicity, age, educational background and employment status.

Based on a multivariate analysis, rural dwellers (MOR 2.0, p < 0.01), the 15–24 year-old (MOR 4.3, p = 0.01) and the 35–44 year-old (MOR 3.4, p = 0.04) were more likely to believe that smoking does not increase one’s chances of getting cardiovascular disease.

### Domain 3: knowledge and attitude regarding the risks of second-hand smoking

Approximately 15.5% of participants did not object to people smoking in their house. This finding was very strongly associated with males (MOR 4.1, p < 0.01). It was also associated with rural dwelling (MOR 1.4, p < 0.01), primary level of education or less (MOR 1.6, p = 0.02), and findings were borderline significant for the unemployed (MOR 1.5; CI: 1.0-2.1; p = 0.05).

More than 98% of participants considered it important to have a smoke-free work place. When disaggregated by gender and education, males and those with least education were more likely not to see the need for a smoke-free workplace (MOR 4.4, p < 0.01 and MOR 3.4, p = 0.03 respectively) (data not shown).

### Domain 4: healthcare counselling on tobacco smoking

Approximately 36.2% of participants responded that a healthcare worker had talked to them about the harms of smoking. Females (MOR 1.3, p < 0.01), rural dwellers (MOR 1.6, p < 0.01), participants aged between 45–64 years-old (MOR 1.9, p < 0.01), and the students (MOR 1.7, p < 0.01) had a higher chance of having been advised about the harms of smoking by a healthcare worker Table 4.

**Table 4:** Healthcare counselling on tobacco smoking within the Mongolian population; 2010

**Have been advised by healthcare workers**	MOR**	**p-value**
**Gender**		
Male	1.0	-
Female	1.3 (1.2-1.6)	<0.01
**Urbanicity**		
Urban	1.0	-
Rural	1.6 (1.3-1.8)	<0.01
**Age**		
15-24	1.0	-
25-34	0.9 (0.7-1.2)	0.5
35-44	1.2 (0.9-1.6)	0.2
45-54	1.9 (1.4-2.5)	<0.01
55-64	1.9 (1.4-2.6)	<0.01
**Education**		
Primary	1.0	-
Secondary	1.2 (0.9-1.7)	0.2
Tertiary	1.4 (1.0-1.9)	0.08
**Employment**		
Student	1.7 (1.3-2.3)	<0.01
Retired/Home	0.7 (0.5-0.9)	<0.01
Unemployed	0.8 (0.6-1.0)	0.02
Employed	1.0	-

**Multivariate Odds Ratio (MOR) adjusted for gender, urbanicity, age, educational background and employment status.

## Discussion

This KAP survey confirms the STEPS findings on the prevalence of tobacco smoking in Mongolia, reflecting high levels of tobacco smoking among Mongolian men. Indeed, 46.3% of Mongolian men were found to be current smokers as part of the KAP survey compared to 48% in the 2009 STEP survey, and compared to 51.4% and 61.3% of men in the neighbouring countries, respectively China and Indonesia in 2009 [8]. This contrasts with only 6.8% of females smoking in Mongolia.

The very high prevalence of smoking among Mongolian males, contrasting females, supports existing evidence of the influence of gender on tobacco smoking. Indeed, the prevalence of tobacco smoking in some countries, especially in Asia, remains strongly associated with the male gender. Historically, smoking is a way of assuming the gender role of masculinity and the lower prevalence of smoking among females reflects a lower level of social acceptability of this behaviour for women [9-11]. This gender role effect is further reflected in this KAP survey, finding a relation between males and certain attitudes toward smoking. Examples include males being more tolerant towards people smoking inside their house and being less inclined to believing in the necessity of a smoke-free working environment. Although the data is lacking in this survey, other pathways through which tobacco smoking could be less prevalent among women in Mongolia are the matters of fewer financial means and the larger amount of daily time spent in the vicinity of babies and children whom women might want to spare from the harmful effects of second-hand smoking.

This KAP survey also revealed that the prevalence of tobacco smoking peaked for individuals aged 45–54 years at 28%, with a prevalence of youth smoking of about 15%. The fact that youth smoking prevalence is almost half of that in 45–54 year-olds might indicate that some measures are preventing tobacco smoking rates among Mongolian youth; however these measures are insufficient given a high prevalence of youth smoking of 15.2% with around 28% of male youth and around 4% of female youth smoking.

Tobacco smoking was moreover particularly associated with urban dwelling and unemployment. These findings support the idea that urbanization itself as a social phenomenon might directly lead to an increase in tobacco smoking. Moreover, urbanization might lead to a higher prevalence of smoking through pathways such as increased revenues, more time spent in social activities, increased exposure to tobacco products and ads, and the desire to emulate the successful Western urban dweller that has traditionally been portrayed as a smoker.

Although the level of awareness about the health risks of smoking was generally very high among the population in this KAP survey, the more educated and the older participants tended to have a higher level of awareness of the health risks of tobacco smoking. However, no significant association was observed between level of education and smoking prevalence. Combined, these findings support the idea that level of awareness alone might have little impact on the healthy behavioural outcomes in a population.

Finally, in terms of tobacco-use counselling, women were more likely to have been advised about the harms of smoking as compared to men. This might once again be a consequence of gender role effects with relation to health-seeking behaviours. Women might also be more strongly advised by Mongolian doctors to stop smoking if it is viewed as socially undesirable for women to smoke in Mongolian society, or if it is more socially acceptable for Mongolian doctors to provide women rather than men with advice on changing their habits. On the other hand, men might have been less willing for some reason to report having received advice about the harms of smoking from a healthcare professional.

Healthcare worker advice on smoking cessation has been shown to increase rates of smoking cessations and should be systematically provided to all smokers [12,13].

In light of the existing policies on tobacco consumption and their degree of reinforcement in Mongolia, the findings of this research allows the formulation of further recommendations for Public Health practice with proven efficacy, especially targeting three populations: Mongolian men, urban Mongolians and Mongolian youth (Table 5).

**Table 5:** Translating Mongolian NCD KAP findings into public health practice

**At-risk population**	**Findings from KAP study**	**Suggestions for public health practice**
Mongolian men	High prevalence of smoking; riskier attitudes towards smoking i.e. allowing smoking indoors; and less inclined to acknowledge the necessity of smoke-free working environments.	● Increase and improve taxation on cigarettes and other tobacco products [14-16].
		● Reinforce restrictions on the advertisement of tobacco products.
		● Increase picture health warnings on cigarette packs [14,17].
		● Use gender-sensitive prevention and harm reduction techniques such as counter-advertisement [18].
		● Prohibit smoking in all public spaces and office spaces [19].
		● Increase provision of smoking cessation counselling by healthcare providers.
		● Increase access to free smoking cessation programs, for example quit lines [20].
Urban Mongolians	Higher prevalence of smoking.	● Same measures as for Mongolian men.
Mongolian youth	High prevalence of smoking compared to other countries; less awareness about the health harms associated with smoking.	● Increase the level of early education on the harms of tobacco smoking [21].
		● Reinforce restrictions on the advertisement of tobacco products [22].
		● Prohibit the sale of tobacco on store shelves and the sale of non-tobacco products as tobacco products.
		● Reinforce the ban on selling tobacco to youth under 16 at point of sales.

## Conclusion

This KAP Survey confirms the findings of the 2009 STEPS Survey and complements knowledge around the drivers of smoking in Mongolia.

This survey confirms a high prevalence of smoking in Mongolia, especially among men and urban dwellers, and reveals a concerning prevalence of smoking among Mongolian youth. It also suggests that the Mongolian population has a high level of knowledge overall on the harms related to tobacco smoking; however, some attitudes and practices surrounding tobacco smoking remain harmful, especially so among males.

Identifying males, urban dwellers and Mongolian youth as at risk populations as well as some of the attitudes underlying such risk allows suggestions for improved or new public health measures. The Mongolian population might benefit from these measures in the context of its increasing urbanisation and heavy burden of non-communicable diseases.

## Competing interests

The authors declare that they have no competing interests.

## Authors’ contributions

AD and JN drafted the manuscript and undertook data analysis. AD and OD participated in the design and implementation of the study. All authors read and contributed to the manuscript drafts, including the final manuscript.

## Pre-publication history

The pre-publication history for this paper can be accessed here:

http://www.biomedcentral.com/1471-2458/14/213/prepub
